# Changing publication practices and the typification of the journal article in science and technology studies

**DOI:** 10.1177/03063127221110623

**Published:** 2022-07-28

**Authors:** Wolfgang Kaltenbrunner, Kean Birch, Thed van Leeuwen, Maria Amuchastegui

**Affiliations:** 1 Leiden University, Leiden, The Netherlands; 2 York University, Toronto, ON, Canada

**Keywords:** publishing practices, scholarly communication, journals, science and technology studies, doability

## Abstract

In this article, we study the development of the STS journal article format since the 1980s. Our analysis is based on quantitative data that suggest that the diversity of various journal publication types has diminished over the past four decades, while the format of research articles has become increasingly typified. We contextualize these historical shifts in qualitative terms, drawing on a set of 76 interviews with STS scholars and other stakeholders in scholarly publishing. Here, we first portray the STS publication culture of the 1980s and early 1990s. We then contrast this with an analysis of publishing practices today, which are characterized by a much more structured research process that is largely organized around the production of typified journal articles. Whereas earlier studies have often emphasized the importance of rhetorical persuasion strategies as drivers in the development of scholarly communication formats, our analysis highlights a complementary and historically novel set of shaping factors, namely, increasingly quantified research (self-)assessment practices in the context of a projectification of academic life. We argue that reliance on a highly structured publication format is a distinct strategy for making STS scholarship ‘doable’ in the sense of facilitating the planning ability and daily conduct of research across a variety of levels – including the writing process, collaboration with peers, attracting funding, and interaction with journals. We conclude by reflecting on the advantages and downsides of the typification of journal articles for STS.

How have journal publication formats changed in science and technology studies (STS) over the past four decades? And to the extent that they have changed, why? Traditional functionalist and positivist perspectives suggest that epistemic requirements determine the principles according to which researchers communicate their findings among each other. Yet, STS repeatedly documented how epistemic concerns do not exist in abstract forms, but always are bound up with contingent histories of scholarly communication practices.

Shapin and Schaffer (1985), for example, charted the emergence of a new understanding of scientific expertise in 17th-century England, based on the public witnessing of experiments. In this account, Robert Boyle was successful in challenging the authority of natural philosophy not simply because of the inherent superiority of his experimental program, but because he combined an ideal of interpersonal trust among scholars with elaborately orchestrated material demonstrations and literary strategies for scholarly communication. The latter consisted in giving rhetorically convincing accounts of experiments, which enrolled readers as virtual witnesses of their outcomes. In a more recent historical account of the development of the scientific journal in France and Britain, Csiszar (2018) problematizes the notion of a singular scientific publication format that allegedly emerged in a fully-fledged form around 1700. Instead, he draws attention to the original multiplicity of forms of journal publishing and the gradual reduction of this variety, especially in the course of the 19th century. Csiszar (2018) argues that the reduction was driven by a complex interplay of competing interests among different types of scientific publishers, attempts to order the growing production of journal articles through bibliographic technologies, and the growing identification of professionalized scientific work with authorship of journal publications.

There is also a wealth of detailed analyses of the shaping of more contemporary publication formats. Bazerman (1988) provides case studies of how, in an effort to gain credibility and prestige, 20th-century US psychologists and political scientists increasingly imitated the standardized publication formats of the natural sciences. Making reference to Bazerman (1988), Abbott and Barman (1997) analyze the rhetorical composition of articles published in the *American Journal of Sociology* between 1895 and 1965. They conclude that, while some standardization of the format is going on, it is not a uniform development, which the authors speculate could point to competing definitions of persuasive reporting over time. More recently, Strang & Siler (2017) observe a trend away from more essayistic writing to experimental reports as the dominant article format in the journal *Administrative Science Quarterly*. Their argument emphasizes the importance of rhetorical strategies used by scholars within their own community as a main shaping factor, born out of the growing difficulty to defend knowledge claims in an increasingly competitive field.

In this article, we focus on a complementary yet understudied and historically novel set of factors in shaping publication formats in STS itself, namely, increasingly quantified research (self-)assessment practices in the context of a projectification of academic life (Sigl, 2016; Torka, 2009). Previous research has shown how concerns with maximizing publication output and anticipated citation impact in a variety of fields have led to a profound reshaping of scientific workflows, for example, in terms of how scientists decide which research projects to pick, which collaborations to seek out, and when research projects should be considered completed (Müller & de Rijcke, 2017; Rushforth & de Rijcke, 2015). Müller (2014) argues that postdocs in the life sciences compete for advancement to a group leader position by subordinating all of their activities to a strict ‘impact per time’ calculus. At the same time, there has been hardly any attention to how practices of this sort affect publication formats themselves in the long run, at best correlating them with ‘salami slicing’ and quasi-versioning of articles (Casati, 2010; Cronin, 2013) and abandonment of activities that don’t lead to high-impact journal articles (Butler, 2007; Laudel & Gläser, 2006). However, a precondition for the productivist approach highlighted by these studies would seem to be a relatively uniform understanding of publication formats in the first place, to allow them to act as a metric around which other activities can be planned and economized.

## Publication formats and the relative doability of research

In some influential publications, Fujimura (1987, 1992) proposes that the production of academic knowledge requires the creation of temporary alignments between different organizational levels, each of which pose distinct forms of uncertainty that threaten the successful conduct of research (Fochler, 2016; Fujimura, 1987, 1992; Kaltenbrunner, 2020; Sigl, 2016). A first level of organization is that of particular research projects. Here, uncertainties arise due to the unpredictability of research itself. Researchers are, moreover, embedded in broader institutional contexts such as department and universities. Their projects must be accommodated in these overarching contexts, both practically, in terms of having to mobilize institutional resources and balancing competing demands on research time (e.g. teaching obligations), and also in terms of requirements related to tenure or evaluation. In addition, there are external contexts and audiences to which research must be made relevant in some way. This includes attracting grants, and thus the interest of funders, to be able to carry out projects in the first place. It also requires publishing research in suitable outlets, legitimating it vis-à-vis peers in an academic community. Research is doable when these diverse levels of organization can be made to align.

The complexity of achieving such alignment has increased in recent decades. Many academic fields have faced new formal and informal demands regarding the productivity and accountability of researchers, as well as an increasingly competitive job market leading to a growing number of academics spending more time in temporary contracts (Goastellec et al., 2013; Teichler et al., 2013; Waaijer et al., 2017).

In the specific case of STS, an additional challenge stems from the changing funding structure of the field: Besides growing dependence on external funding in general (Lepori et al., 2007), large-scale thematic grant programs such as those around genomics in the UK in the 1990s and 2000s or Responsible Research and Innovation in Europe since roughly 2010 have exerted an increasingly important steering influence on STS research practices, research agendas, and collaboration structures. Yet another factor is the sheer growth of the field and its differentiation into many diverse conceptual traditions and niches, which makes it increasingly unclear for whom one is or should be writing.

Previous scholarship has usually focused on how packaging of methods and ad-hoc work to ensure and repair alignment between organizational levels facilitate making research doable in the face of such diverse pressures (Fujimura, 1987, 1992; Kaltenbrunner, 2020). Turning originally complex and decision-laden sequences of scientific tasks into black-boxed methods removes degrees of freedom from daily research practice that scientists no longer have to worry about and around which other tasks can be more flexibly organized.

Publication formats connect all of the above-mentioned levels of research organization in specific ways. This becomes perhaps especially clear to early career scholars when they are first initiated into publishing and find themselves confronted with a range of fundamental questions: With a limited period of funded research time available, which types of publication should they pick to ensure that research becomes visible to relevant audiences, is useful for career development, and will help in securing follow-up funding? (Nästesjö, 2021) How should they frame research questions, present research methods, and structure arguments to appeal to the various intended audiences? (Swales, 2004) In the case of collaborative writing projects, how should they deal with questions of shared authorship and division of labor with supervisors and colleagues?

The complexity of such decisions is directly related to the relative degree of homogeneity of publishing formats in a given intellectual formation (Whitley, 2000). In emerging fields of study, journal publications are often characterized by lack of agreement on how research should be presented, requiring authors to make cases not just for particular claims, but also for the framings of their argument. Moreover, in interdisciplinary areas of study, it can be difficult for scholars to identify dedicated journals for their work in the first place. Authors may have to circulate their research in outlets of other, more consolidated, fields, but at the expense of having to conform to their diverse expectations and epistemic standards. There are also many areas in the humanities and social sciences where monographs are still considered important. Aside from developing a coherent prose narrative over two hundred pages or more without a readily pre-structured format to rely on, a special challenge here is to accommodate a longer-term book project with the need for scholars to demonstrate consistent productivity over time. In short, where publishing formats are heterogeneous and not very standardized, every single publication forces scholars to make a multitude of far-reaching individual choices.

## Typification as a way of facilitating doability

A high degree of agreement on the format of publications reduces uncertainty for authors and enables various forms of efficiency that are particularly helpful in project-based work. Today’s grant applications generally require researchers to specify intended research outputs, most importantly in the shape of high-impact journal articles. The above-cited research on publishing practices in the life sciences has shown how scientists organize their workflows with such intended output in mind, using them as milestones around which various other tasks can be planned. Scientists can try to gauge the minimum of substantial results necessary to warrant submission to particular journals and they can use the widely agreed-upon formal division of articles into elements such as methods and findings to divide tasks among collaborating authors (Müller, 2014; Müller & de Rijcke, 2017; Rushforth & de Rijcke, 2015). Following a dominant publication format also ensures a degree of common ground between authors and prospective readers, which is especially helpful in growing and epistemically fragmenting fields, where shared intellectual context is no longer ensured through personal acquaintance.

However, typification of publication format affects the content of presented research. Scholarship on sociotechnical standards has highlighted the productive epistemic potential of agreed-upon data formats and methods in the sciences, which facilitate reuse of resources and thereby concentrate attention around shared intellectual agendas (Borgman, 2007; Bowker & Star, 1999; Edwards et al., 2011). A publication culture based on imitation, and thereby entrenchment, of a particular article format can promote a similar effect. It presents researchers with textual models for how to summarize and interpret earlier findings, theories, and concepts (Bazerman, 1988; Whitley, 2000), enabling them to mobilize previous research contributions as epistemic resources. This can be done in different ways. On one hand, previous work can be more easily seen as a ‘body of literature’ that is inevitably characterized by ‘gaps’, for example, in the shape of empirical blind spots. On the other hand, concepts and theories can be more easily framed as transportable analytical frameworks that can be ‘applied’ to new empirical problems. Not least, greater homogeneity in publishing formats can make it easier to partition individual contributions around a publishable unit. This principle has been described as typical of maturing fields, where competition often prompts researchers to aim for narrowly defined contributions that can be more easily defended (Bazerman, 1988; Ziman, 2000).

Research on the history of academic publication formats has often tended to highlight how epistemic concerns are bound up with rhetorical strategies to convince peers or external audiences of one’s claims. Our analytical focus is compatible with such accounts, but suggests that rhetoric and persuasion are merely an element in an overarching concern of authors with doability. We emphasize how publication formats are shaped by a more fundamental interest in removing degrees of freedom from research practice, which frees up attention for authors to worry about other challenges in aligning contexts and constraints. To put it bluntly, the less researchers have to think about how to write and publish, the better they can focus on attracting funds, engaging in collaboration, making their work relevant to non-academic audiences and simply publishing more. If such doability techniques are commonly used on a large scale, say, on the level of a field, it will promote the typification of a publishing format – the notion of a typical article format in a field emerges or is consolidated.

So far, we have focused on the practical benefits of a homogeneous publishing format for authors. But are there any disadvantages? Arguably, the most obvious disadvantage is the risk of publications becoming overly formulaic. We do not wish to suggest that a relatively uniform publication culture works against originality in an undifferentiated sense – clearly, there is a lot of vibrant research in many fields that is written in otherwise typical formats. Yet the homogenization of publishing formats and the removal of degrees of freedom it implies can be theorized to systematically limit or foreclose certain sources of intellectual originality.

For example, a particularly dominant way of interpreting and using theories and concepts will promote their packaging into transportable epistemic resources. While black-boxing of successful theoretical work is an inevitable part of scholarship (Latour, 1987; Rheinberger, 1997), a pervasive concern of authors with maximizing the doability of their research can accelerate this process and unduly entrench particular conceptual perspectives. The convention of carefully partitioning contributions into narrowly defined arguments in turn can degenerate from mere frugality to salami-slicing, since there is no sharp boundary between these practices (Ioannidis, 2016). At worst, a highly typified format also makes it possible for authors to engage in a form of writing that primarily consists in imitating surface structures of a typical article. Literature, for example, can be cited for the sake of filling in a section and mobilizing rhetorical support for a claim, however, without good (epistemic) reasons for doing so. For individual authors, the benefits of their doability strategies will tend to outweigh such downsides. Yet, we expect that especially in interpretive fields such as STS, in which ideals of individual creativity, a plurality of intellectual perspectives and more literary forms of expression are widely considered important, the rise of typified publication formats may give rise to collective discontent with the longer-term problems of ‘formulaic writing’.

## Data and methods

This article is based on three forms of empirical material. First, we draw on a scientometric analysis of eighteen STS journals that have gradually been included into the Web of Science (WoS). The purpose of this analysis is to identify overarching developments in STS journal publishing: changes in formal features such as page lengths and number of references, as well as co-authorship patterns. The methodological details and a complete list of the journals are described separately in Supplemental Appendix 2.

Second, we offer a partial content analysis of all articles for two sample periods (1990–1994 and 2015–2019) published in four of the oldest journals typically associated with STS: *Social Studies of Science* (*SSS*); *Science, Technology, & Human Values* (*ST&HV*); *Minerva*; and *Science as Culture* (*SaC*). The purpose of this comparison is twofold. We trace the changing distribution of different types of publications (designated as ‘articles’, ‘comments’, ‘discussion pieces’, ‘book review’ and so on) over time, which is one way of gauging the relative diversity of STS journal publishing. We also trace quantitative and qualitative changes in specific formal features of publications designated as ‘research articles’: how articles introduce their arguments (either by making a case for their inherent interest or by creating explicit groundings in academic literature through bibliographic references in the first five pages), and the relative share of articles that are based on empirical work as opposed to purely conceptual or literature-based publications. For articles based on empirical work, we indicate whether there is an explicitly designated data and methods section and what types of method were predominantly used to collect material (archival/historical work, document analysis, ethnographic work, and interviews). These features can partly be seen as indicative of a process of formalization of the research article. Bibliographic positioning of a contribution is typically associated with less essayistic and more formal types of writing, as is the grounding of research articles in explicitly described methodological approaches (Bazerman, 1988; Strang & Siler, 2017). A comparison of these features over two time periods allows us to determine to what extent differences in format diminish over time, thus providing a quantitative indication of the degree of typification of articles in the four STS journals.

To contextualize these findings, we draw on a set of 74 interviews with STS scholars from different career stages and in different functions in the STS publishing system (editors-in-chief and managing editors, editorial board members, referees and, of course, published authors) as well as two interviews with representatives of commercial publishers. We interviewed twelve scholars who were or had been in editor-in-chief or managing editor roles. Many other informants were on the editorial boards of one or more journals. We conducted the interviews between November 2019 and June 2020; the interview questions covered the informants’ perception of various types of changes in the STS publishing landscape in recent decades (such as the emergence of the journal impact factor and other metrics, competition for publication space, digitization of publishing, and the relation between journals and monograph publishing) as well as the implications of these changes for research practices and academic careers. For scholars in an editorial position, we inquired about how they perceive their special role in the context of the previously discussed transitions in publishing. All interviews were transcribed in full. Using the Nvivo qualitative data analysis software package, we coded the transcripts according to iteratively refined categories that focus on diverse aspects of the publishing process, for example, ‘publishing formats and collaboration’, ‘picking journals’, ‘writing process’, and ‘developments in STS theory, concepts, methods’. Because STS is a relatively small field, to ensure anonymization we provide no details about the demographics, location, or institutional affiliation of any informants when quoting from interviews.

## Reduction of diversity in STS journal publishing

**Figure 1.**
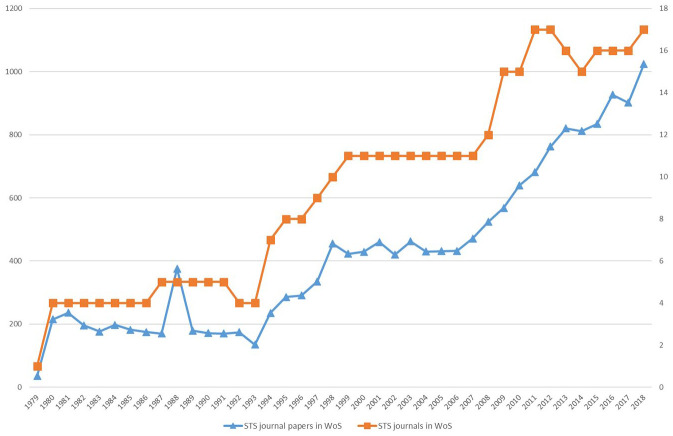
Production of articles and number of journals.

Our scientometric data shows that the production of articles across the 18 selected STS journals indexed in WoS has steadily increased since the 1980s. Figure 1 charts both the aggregate number of STS journal publications and the number of STS journals in WoS. We see periods of pronounced growth in the mid-1990s and since the mid-2000s.

**Figure 2.**
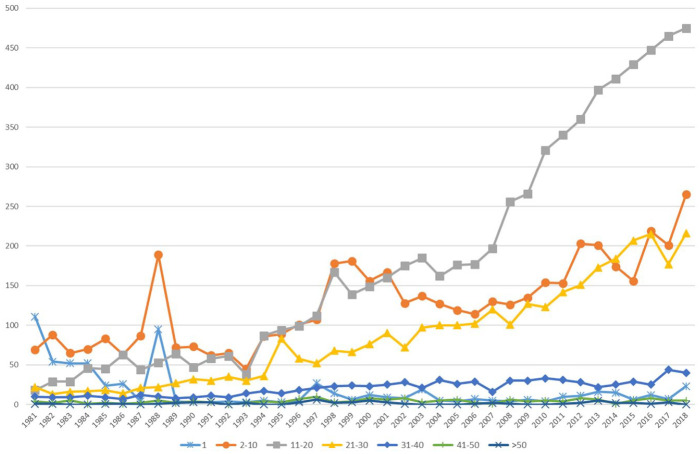
Page length of publications (18 journals).

**Figure 3.**
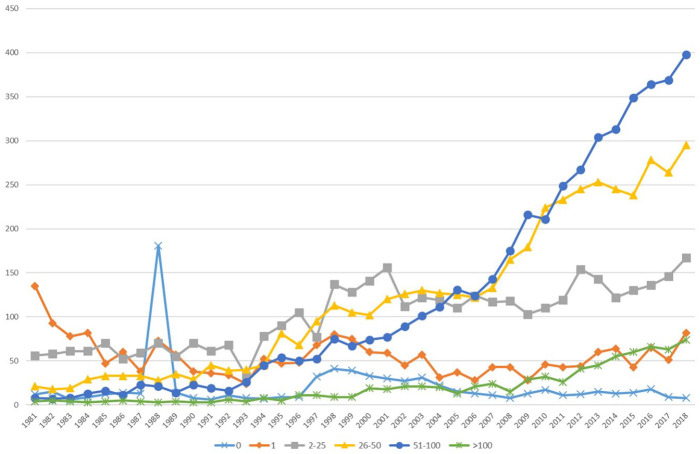
Number of references per publication (18 journals).

Figures 2 and 3 show trends in page length and number of references. Initially, page length varies, without any length being particularly dominant. This is matched by heterogeneous numbers of references, which vary between as few as 1 and as many as 100. This suggests a balance of different types of publications (e.g. research articles, comments, discussion pieces, and book reviews) and/or diverging understandings of what particular publication types should look like. Later, namely from the mid-2000s onward, the number of papers with 11–20 pages increases sharply relative to the others. Papers in the range of 21–30 and 2–10 also increase, albeit more moderately. Extremely long papers become comparatively marginal. This development is accompanied by an increase in the number of papers with 26–100 references. These developments can potentially be seen as pointing to some form of decrease of diversity of article types and the growing typification of publishing formats.

**Figure 4.**
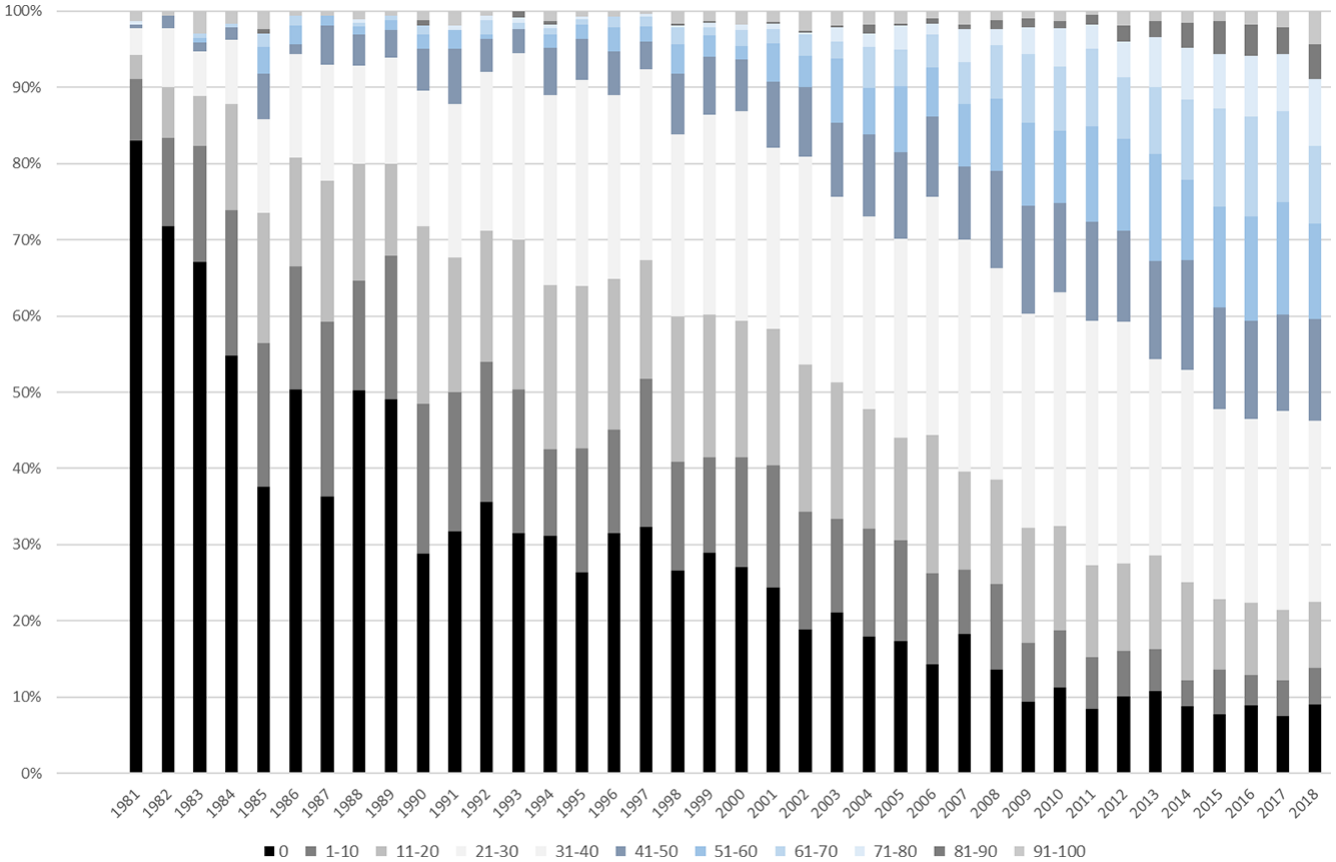
Citation targets.

There are two more aggregate trends that are at least compatible with such an interpretation. Figure 4 is a visualization of the changing proportion of references by STS articles to other WoS publications over time. The different color shades of the bars here indicate distinct categories of reliance on WoS as a source of references. For example, black denotes the proportion of articles that have no references to WoS publications at all and dark blue indicates the proportion of articles whose reference lists consist of 41%–50% of WoS publications. The figure shows that WoS articles initially were hardly cited at all. Among all STS journal publications in WoS in 1990, some 30% have no references to other WoS journal articles whatsoever. In subsequent years, however, we observe a reversal of this trend, with the share of publications per year referencing literature indexed in WoS increasing steadily. From 2015 onwards, roughly 40% of the articles refer predominantly to other WoS publications (that is, more than half of the literature they cite is indexed in WoS). This illustrates the growing importance of indexed journal articles that is likely to be at the expense of citations to books, book chapters, and other types of output. Journal publishing thus becomes more self-referential in the sense that authors publishing articles in WoS-indexed STS journals increasingly use other WoS publications as their main literature base – and thus potentially as a model for their own writing.

**Figure 5.**
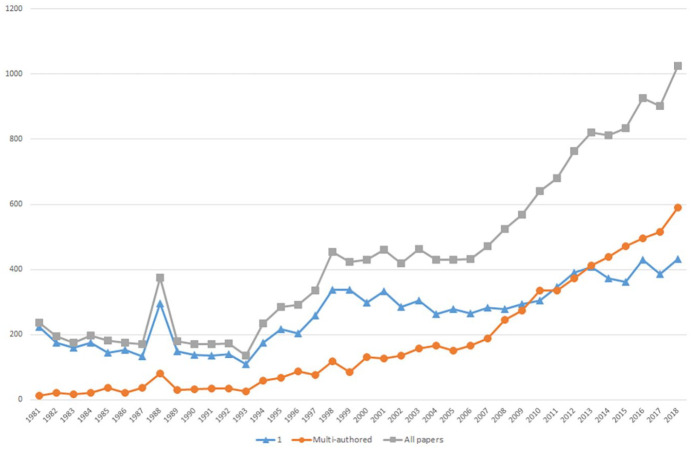
Authorship patterns.

**Figure 6.**
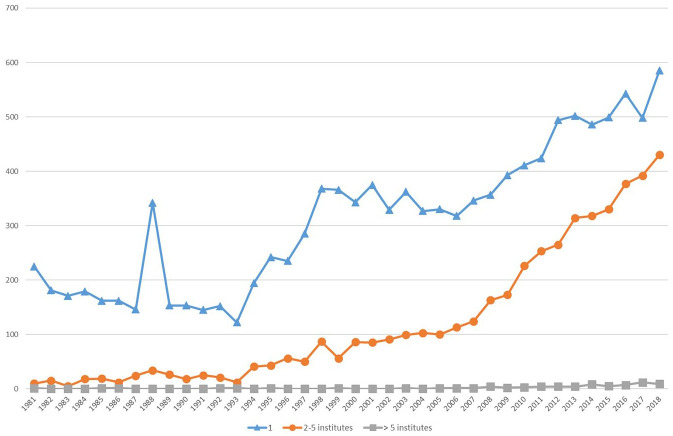
Authorship across multiple institutions.

Journal articles also become an increasingly collaborative form of communication over time. As shown in Figure 5, the number of multi-authored papers begins to surpass single-authored ones around 2009, although single-authorship remains common. It is roughly around the same time that the share of papers by authors based at two or more different institutions significantly increases, as shown in Figure 6. Collaboration in writing apparently occurs increasingly across physical distance, which is typically easier where co-authors agree on publishing formats (Whitley, 2000).

To further interrogate whether there is indeed a historical decrease in diversity of publication formats in STS journals, we now take a more detailed look at the types of articles published in particular STS journals over time. Here, we focus on four core journals that were established before 1990 and thus can be used for a longitudinal comparison: *SSS, ST&HV, Minerva*, and *SaC* (see Supplemental Table 1 in Appendix 1).

In the case of *SSS*, every issue in the first half of the 1990s contains roughly as many research articles as there are ‘comments’, ‘notes and letters’, and ‘responses and replies’. If we include book reviews, the number of non-article publications outweighs articles. Yet, in the five years between 2015 and 2019, non-article publications have decreased significantly, while the number of articles has grown significantly.

*ST&HV* follows a similar trend. Articles are the most common publication type in the first half of the 1990s, but the proportion of ‘comments’, ‘responses’, ‘letters to the editor’, and discussion pieces is still at around 40% per annual volume and never dips below 20% of that number. From 2015 onward, however, all non-article publications become marginal. The only noteworthy exception are four comments published in 2017. These are part of a special issue, however, where publication formats where arguably agreed upon among contributors on a one-by-one basis.

The journal *Minerva* illustrates an even clearer reduction in diversity of publication types. For the first half of the 1990s, there are between 12 and 16 research articles per annual volume. While articles always outweigh the combined number of publications categorized as ‘documents and reports’, the proportion of the latter ranges between 33% and 87.5% per annual volume. If reviews are added, the numbers are almost even. From 2015 onward, only research articles and a few books reviews are published. The two ‘comments’ are again from a special issue.

At first sight, *SaC* seems to follow a different trend. In the early 1990s, all articles aside from review essays are categorized as articles – there are no ‘comments’, ‘responses and replies’, etc. However, as we will elaborate below, the article format in *SaC* is in itself particularly heterogeneous in the 1990s, encompassing publications that would elsewhere be categorized as ‘discussion pieces’ or essays. Moreover, there are 10 explicitly designated ‘comments’ and ‘discussion pieces’ in the time between 2015 and 2019, but, again, these are consistently part of special issues and arguably invited by guest editors.

In summary, then, there is a trend toward reduced variety of publication types across journals, with essay-like and dialogical formats becoming limited to special issues that warrant personal interaction among authors. It should also be noted that these increasingly rare non-article formats are themselves very diverse in format and content. They contain not only replies and reflections on, say, an article of a colleague, but also feature more experimental types of writing such as poems and dramatic dialogues.

What can we say about changes within the format of research articles more narrowly? Is there a typification of the format going on, in the sense of an increasingly singular understanding of what an STS research article should look like across different journals? To answer this question, we offer a partial content analysis of all articles published in the same four journals for two separate periods of time, 1990–1994 and 2015–2019 (see Supplemental Table 2 in Appendix 1).

In *SSS*, a research article format characterized by a specific set of features is already well-established in the early 1990s and changes only in the sense of these features becoming more pronounced over time. A clear majority of articles published in the first half of the 1990s introduce their topics by explicit bibliographic references to scholarly literature from the sociology, philosophy, or history of science that leads them to outline some distinctly academic research interest for STS. This is either framed in terms of a gap in the body of existing research (‘In the last decade, the study of laboratory practices has emerged as a new line of enquiry in social studies of science. … However, Latour does not specify the role of non-human resources’.) or as an application of an existing theoretical approach or concept to analyze new empirical material (‘In this article, we apply the actor-network perspective to study …’). At the same time, articles cite other literature exclusively in footnotes, rather than via in-text references. This creates room for authors to digress and elaborate on the main text, giving articles a somewhat essayistic character. A large proportion of articles is based on empirical data, although the overall share of publications with an explicitly designated methods section is low at around 20%. Papers based on historical or archival sources and other documents at this time are more prevalent than work based on interviews, ethnography, and quantitative analysis. By contrast, in the second half of the 2010s, the share of articles introducing their argument by reference to other literature reaches nearly 100%, similar to the proportion of articles based on empirical data. The referencing style switches to in-text reference, thus reducing digressions, while the proportion of articles with an explicit methods section increases to roughly 40% per year on average. The proportion of research being based on ethnographic work or interviews increases, whereas historical studies become less common.

By contrast, *ST&HV* articles in the early 1990s are not yet as consistently introduced by reference to academic literature and there is a relatively large proportion of articles (ca. 40%) without empirical material, that is, articles of a more conceptual nature. In the late 2010s, however, publications are predominantly introduced by reference to previous literature. Simultaneously, the empirically based article becomes the absolute norm, with a growing share of articles containing also an explicit method section (from 15% to 45%). One might say that articles in *ST&HV* increasingly approximate the model of *SSS*, also in terms of the distribution of methods used.

The trend toward a more typified research article format is even more pronounced in *Minerva* and *SaC*. Opening sections of articles in *Minerva* from 1990 to 1994 hardly ever introduce their topic by reference to gaps in academic literature. Instead, authors open their articles either with an anecdote or simply jump into the topic, thus presupposing that their contribution is of sufficient general interest to an ill-defined audience. Many articles also start with a thesis or broadly defined question that is then explored or defended (‘Can modern technology and democracy coexist?’ ‘Universities are in decline because academics have failed to defend their autonomy’). Like *SSS* in the early 1990s, articles cite other literature exclusively in footnotes, which makes it harder for readers to immediately gauge the embedding of an argument in scholarly discourse and simultaneously creates room for digression. Between 60% and 80% of all articles per year are based on empirical data, although explicitly designed methods sections are extremely rare. Most articles from this period are based on archival work or other historical sources, on document analysis and, to a lesser extent, on interviews. Ethnography and participant observation are completely absent.

In *SaC* articles of the early 1990s, references to academic literature in opening sections are scarce, suggesting more essayistic writing. The few existing references as well as the implicit conceptual background for many contributions are predominantly drawn from a Marxist tradition of critique of science, with the Frankfurt School and figures such as Raymond Williams featuring prominently. Explicit methods sections are practically non-existent, although roughly half of all articles appear to be based on the analysis of empirical material. Most commonly, authors draw on some form of analysis of documents or material culture. There are also various articles previously published as magazine articles or even transcripts of radio transmissions.

By the second half of the 2010s, the differences between articles published in *SaC* and *Minerva* compared to those in *SSS* and *ST&HV* have diminished significantly. As such, they now consistently introduce their topics by outlining an academic research demand or by mobilizing an established conceptual framework from a distinct body of STS literature; they increasingly outline methods of data collection and/or analysis in a dedicated section; and they predominantly draw on empirical data that is now much more commonly generated by interviews or ethnographic work. *Minerva* also adopts an in-text referencing style.

We conclude that article formats across the four journals have become significantly more homogeneous over the course of 30 years.

## STS journal publishing in the 1980s and early 1990s

To contextualize the above quantitative findings and throw into relief the recent emergence of a dominant typified article format, we first outline recurring perceptions of STS publishing practices in the 1980s and early 1990s from our interview material. Established researchers we interviewed agreed that the main challenges to making their work doable were connected to the changing contexts of the field of STS. A key difficulty was the attempt to establish a wholly new field of research while also pursuing an institutional career in an academic landscape dominated by more established disciplines such as sociology, history, and philosophy. In terms of publishing practices, writing monographs was a much more common practice in the early decades of STS, with monographs being widely considered the main indication of intellectual independence (#8, #50, #57, and #59). While concern with publishing articles did exist, quantity and regularity of publications was much less of a concern than is the case today. A telling indication is that authors in the 1980s appear to have been much more concerned with the possibility of damaging their reputation by circulating or submitting underdeveloped papers and manuscripts. PhD students, for example, were generally not expected to publish, because there was a sense that publishing precociously could be harmful for their career. One informant stated:

I graduated with my PhD in [the late 1980s]. The idea that I would publish anything when I was still a graduate student would have been considered absurd and presumptuous. Certainly, I didn’t know enough to go publish anything. Now, as you probably are aware, it’s expected that if you get a PhD and go out in the job market, you got to have publications. … I didn’t have anything interesting enough to say when I was 30 years old, and if I did, I didn’t know how to say it because I didn’t know how to write well enough. (#25)

Another informant explained their perception at the time that publishing many journal articles would be ‘crude’.

I mean, when I was in grad school, it struck me at the time – just looking back, I can sort of remember feeling this way – that it seemed kind of crude to publish as much as people publish now. … And, you know, it was not common or encouraged for PhD students to publish articles. And professors even, I mean, if they published one article every three years, that would be something. There was more of an emphasis on books, and I’d say every five to eight years a book or so, and just not this article treadmill. (#59)

Informants also indicated that the connection between research activities and intended outputs was significantly looser in terms of prospective planning. While scholars aimed to publish journal articles next to their longer-term monograph projects, the exact format, outlet and type of articles often developed gradually in the course of research activities that scholars pursued regardless. Our interviews feature a number of accounts by now-established STS scholars who described the early years of the emerging field as lacking accepted standards regarding publishing outlets they should aim for. *Social Studies of Science*, before 1975 titled *Science Studies*, had been in existence since 1970, but there were otherwise few obvious journals to aim for. Self-identified science studies scholars would often target other interdisciplinary journals or philosophy of science journals and they would usually come across such outlets by physically browsing their university libraries. One of the informants described their approach to publishing around 1980 as follows:

[A]t that point, there really weren’t STS places to publish and so what you would do would be to look for cross-disciplinary journals. … I used to go across to the library and I’d see a journal [at the intersection of natural sciences and engineering]. Okay, that sounds great because that’s cross-disciplinary. I think I’ll do something for [this journal]. And no one had anything to say about that. … And I always remember having a deliberate policy of sort of moving around between journals as much as possible because I thought, well … – next time will be a completely different place. It was quite haphazard, honestly. Well, I didn’t spend … It was certainly very hard to think strategically about those things. (#50)

Yet, while identifying suitable journals can be called haphazard, authors commonly invested time in trying to understand the profile of the journals they came across and crafted their submissions accordingly. One of our informants (#16) recounted an anecdote where a colleague wished to submit a manuscript to a specific journal but could not get hold of author instructions. Unable to reach the editors, the colleague decided to approach the local embassy of the country where the journal was published, with a request for making contact with the editors. Simply submitting an article in the hope that mismatches in terms of scope, format, and style could be worked out later, apparently, was uncommon.

The few dedicated STS journals that did exist were, in turn, read by a small community, and the volume of literature published was such that individuals could still have overviews of all or most of it. Several informants described how they would consult individual issues when they came out (#50 and #72), often reading them from cover to cover to keep themselves up to date. This combination of a small-sized community and a mere handful of dedicated journals gave the latter a generalist character. Accounting for the many ‘comments’ and ‘discussion pieces’ identified above, journals such as *Social Studies of Science* provided a forum for scholars from diverse theoretical traditions – Marxist critique of science, actor network theory, sociology of science in the vein of either Robert K Merton or the Edinburgh School – who would take competing positions on a limited set of research questions surrounding the social construction of science and technology. STS journals were run almost like small intellectual clubs, in the sense that prominent editorial figures took significant leeway in publication decisions and in mediating communication between authors and reviewers (#72 and #16). It must also be noted that women were severely underrepresented until the 1990s, thus effectively rendering early STS a male discipline.

A hallmark of the STS publication culture of the 1980s and early 1990s was, moreover, an interest in experimental writing (#8, #10, #29, and #50). Some accounts (Mulkay, 1985) proposed stylistic reflexivity as a programmatic consequence of STS scholarship, which they argued could not simply imitate the positivist reporting style of the sciences. Many well-known publications from that period included or consisted of poems and dialogues between different imaginary speakers (e.g. Ashmore, 1989; Mulkay, 1984; Woolgar, 1989). These often ironic elements were carefully crafted to subvert the authoritative ‘voice from nowhere’ characteristic of both scientific papers in the natural sciences and the traditional scholarly monograph. While never wholly uncontested, we propose that such experimental forms were more easily accomplished for two reasons. One is that the community of readers was much smaller, which allowed authors to presuppose the intellectual context necessary to make sense of ironic elements. Another is the relatively lower publication pressure at the time, which gave authors more freedom to explore diverse forms of expression. One informant reflected:

What I really liked about STS in the early days … was precisely the variety in form. That it was possible to be a bit more experimental. … And there’s certainly less of that now or it’s done by particular people in particular places. … as with all experiments, whether it’s in experimental writing, just words on a page or experimental formats in terms of using the kind of digital affordances, it’s hard work. … [A] standard journal article is easier to write than an experimental journal article, I think. … And because of the pressures people are under, people are perhaps more reluctant to take the time that’s needed to experiment, either in writing style or in kind of material realization. And, of course, some of the tools are a lot easier to use, but some things aren’t. And to do those sorts of things well, [it] does take a lot of time and skill and maybe collaboration and all those things that we know are difficult (#10).

In sum, the challenge of making STS research doable in the 1980s and early 1990s was, in some ways, very different from today’s context. While some sources of uncertainty are inherent to the unpredictable nature of research and thus are relatively immutable, distinct challenges at the time were to identify and accommodate the requirements of suitable interdisciplinary journals that would complement longer-term book projects, combine such publishing activity with a research interest in a distinct emerging intellectual formation of STS as well as a distinct form of reputation management to ensure that each and every published article would hold up under the scrutiny of a peer. On the other hand, sheer quantity of journal articles was not an overriding concern and the daily organization of research practice was not built around the production of any particular periodical output besides long-term book projects.

## STS journal publishing today

The portrayal above contrasts with the many mid- and early career STS scholars describe their approach to publishing today, where especially journal publishing is both a major source of worry for scholars as well as a site for preemptively tackling various sources of uncertainty in research. The intended form and quantity of the output is often one of the first considerations in how STS scholars go about research today (#6, #14, #61, and #54). This is configured by the increasingly project-based format of much of STS scholarship, where prospective outputs often have to be defined in explicit terms within the parameters of research funding applications and timeframes. An early career researcher described their recent application experience as follows:

So in my recent grant, I submitted an application to [a funding body], it’s called a [title] project, this is where you get a team of four or five people together to tackle a particular problem … the more specific you can be, the better. We wrote that we aim to publish in high impact STS journals, I think I wrote *ST&HV*, I think I explicitly listed *ST&HV* among that. (#14)

To be clear, it is not necessarily that funders and employing institutions confront scholars with specific formal evaluation requirements regarding their production of journal articles (although some do). Rather, a crucial element influencing their approach seems to be precisely the absence of such requirements or lack of clear criteria, which makes scholars fall back on assumptions about what evaluators and peers might expect of them (see Rushforth & de Rijcke, 2015). Many appear to think that the best preparation for a highly competitive job market combined with ill-defined evaluative requirements are productivity measurements, such as number of publications and citation counts on Google Scholar (#30, #36, #61, #40, and #58). One early career researcher commented:

I’ve been on precarious contracts for a long while. Which means effectively, until about two years ago, I’ve been looking for jobs pretty much every year. … And the one thing you can pretty much guarantee people sitting on interview panels will look at is your Google Scholar profile. And the first thing that jumps off that … is amount of publications, you can guess where they’ve gone to, whether these are decent … journals or not and, also, citations. So, kind of finding ways to evidence what you’re doing on Google Scholar, it does kind of drive where your efforts go, if you’re in a position where you don’t know where your next job is gonna be. …Certainly, there is a pressure to publish more and we like these rapid response journals, you know, we get quicker decisions. … it speeds the process up, but it also creates more pressure, as in, if you get an acceptance, then ‘well, where is the next paper?’ (#40)

Such a productivity-oriented outlook goes along with a significantly more structured research process when compared to earlier periods of STS scholarship. Treating research projects as the production of a typical form of output allows for calculating investments and payoffs, thereby contributing to doability in the daily conduct of research. First, activities can be more easily conceived in terms of a labor-divided undertaking, akin to the laboratory model common in many natural sciences. Several informants outline an implicit economizing view of their research practice, where the latter consists of clearly differentiated tasks that can be divided among members of a team (#14, #36, #53, and #61). A basic distinction that underpins many of our interview accounts is that between grant acquisition on one hand (which is primarily, but not exclusively, a task for established researchers) as well as empirical work and writing (which are often partially or wholly delegated to junior scholars). The following quote suggests that the degree of differentiation of tasks, to some extent, correlates with the size of the grant. The larger the sum of the grant, the more division of labor involved. Interestingly, the particular kind of efficiency that is thereby enabled is sometimes perceived as preventing scholars from more fully developing the most relevant aspects of their work.

But quantity prevails, and it prevails in so many ways. It prevails in [that] you need to get grants, so you need to have publications to get grants. And you need to have, many times, too many grants, and I, certainly, in my career have had too many grants. And so you don’t spend enough time with each grant, you don’t go farther necessarily into what’s useful or interesting. You get some things done and also you don’t – more than that, I think I’ve given responsibility for writing up to postdocs or I have not been able to [unintelligible] because I’ve had to administer grants …. I remember [an older colleague] told me ages ago, very early on in my career, she said … you know you need to resist [grant money], you need to like, not pursue it, not go after it, because you need small amounts of money to do your own research and engage deeply in the topic rather than administering data collection by others. … I, for most of my academic career until seven years ago, I didn’t do any of my own data collection. (#36)

Reliance on a typified article format also creates flexibility on the level of the actual writing process, namely, by decoupling conceptual and empirical elements of an article. Authors commonly treat previous theoretical work in STS as a set of readily available theoretical tools to be applied to distinct empirical objects, which in turn can be connected to (shifting) priorities in science funding (#14, #52, #61, and #48; see Beaulieu et al., 2007; Sovacool & Hess, 2017). A characteristic terminology used in many of our interviews is that of STS theory and concepts as ‘frameworks’ that can be deployed depending on the analytical needs of a given project.

[So] we do really have to pitch all of our research as tackling a genuine societal problem. Now, I do that a little bit strategically. So, I think, okay, once I get the money, then I’m gonna do some really cool STS stuff, which I hope will allow me to publish in those STS journals …. [You] know we have to pitch it more in terms of societal problems rather than academic interests, if that makes sense. … For the application I’ve put forth recently, … I frame this project as tackling a societal issue, I will say that the state of the project will also allow me to bring in some STS frameworks and analysis. And then, when I say what my outputs are, I say I’m gonna do some outputs in medical sociology, some outputs in STS journals, and then some output in maybe interdisciplinary journals aimed at practitioners, and then I have my output for practitioners. (#14)

The tendency to treat theoretical frameworks and concepts as a toolbox for empirical problems – instead of a potential object of research in its own right – does not *per se* work against theoretical innovation, but it does seem to go along with a distinctly incremental understanding of the latter. A common strategy mentioned by several of our informants is to aim for a minimal amount of innovation, namely, by coining a new concept that elaborates on particular theoretical traditions (#9, #24, #52, and #61).

Adherence to a typified article template creates practical advantages for authors in their interaction with journals. Manuscripts written on the basis of a general notion of what a typical STS article should look like can be easily circulated across various outlets in case of initial rejection. Authors can also choose to get reviews from one journal and use them to modify their piece somewhat to resubmit an improved version elsewhere. As a side effect, journals are increasingly distinguished according to the perceived levels of prestige and perhaps minor epistemic specificities, but overall as a homogeneous set of communication channels.

[There] are more places you can submit to, so it almost kind of lowers the stakes of getting a rejection. And if I get one from *Big Data and Society*, maybe I can submit to *Social Media + Society* … that’s not a great way of going about writing quality work necessarily, but it’s an option for people who want to quickly publish papers. (#40)

However, it is not just the common practice of resubmitting papers in identical or near-identical forms to other journals that makes reliance on a standard/typical format convenient for authors. It is also the growth and differentiation of the field that makes such an approach practical. An important context for this perception is the sheer growth in the production of articles and the number of journals since the 1990s, as illustrated in our quantitative analysis. Simultaneously, new outlets have appeared that distinguish themselves through a focus on either particular topics of research or on specific geographical regions. And while these developments make it easier, in principle, for authors to identify suitable outlets compared to the 1980s, it also creates new complexities in the sense that it is more difficult to anticipate what exact publication formats the respective editors and reviewers would find suitable for a given journal. Several informants related that they, on occasion, thought about submitting manuscripts that, for various reasons, diverged from what they perceived to be a typical research article, but ultimately refrained from doing so because they worried that a negative reaction by reviewers might dangerously slow down the publication process (#11 and #61). It also appears that certain changes in the operation of journals play a part in making a typified article format a safer bet. Several informants told us that – whether justified or not – communication with editors who are able to offer informed feedback on the suitability of a given submission has become more cumbersome (#63, #61, and #36), since many journals are run by increasingly large, but less tightly coordinated editorial teams, partly also supported by publisher staff with no knowledge of the field. In other words, the new flexibility afforded by a wider array of publishing outlets is an advantage only if scholars can simultaneously minimize the effort spent on tailoring their submissions to particular journal profiles.

Dominant and increasingly typified as it is, the journal article remains merely one among a range of publication formats used in STS. Our informants agreed, for example, that monographs can still be highly influential and important for career development, especially when published by reputable university presses. At the same time, monographs themselves are often based on journal articles published earlier to prevent a long period without formally accountable publication productivity – widely perceived as a major risk to the sustainability of a career long-term (#50, #62, and #40).

There are also attempts to experiment with alternative formats of journal publishing itself. Several recently founded outlets publish articles that are defined in explicit contradistinction to 8,000–10,000 word research articles, for example, in terms of being significantly shorter and more conceptual in nature. Yet our interviews suggest that the typical article has created a standard of doability and even worth against which other types of articles are measured – often in unfavorable terms. One informant, who is editor of a less well established journal, suggested that shorter conceptual submissions are often leftovers of larger research projects that could not be published in the shape of fully-fledged research articles elsewhere (#1). Another informant suggested that, where referees and/or editors insist that shorter conceptual pieces should make a real contribution, they are actually no less work than a full-length paper, making the investment of time that is required seem questionable.

I’ve got a – yeah, so I did publish a small thing in that [journal]. … [It] is meant to be either two or three thousand words, where you’re meant to take a single novel idea … and apply it once. And that’s the paper. And it was very attractive to me, actually, when I saw it. I thought, oh this is really good, I could imagine myself doing a few of these. The peer review process was tricky. And if you look, they haven’t published many of these. So – and I wouldn’t – because they do, when you put it in for peer review, they do push you to say look, you really do need to come up with a brand new idea here. They really want you to see the intellectual significance, which, I think, it was, and they published it. But you kind of think well, if I’m going to do something that’s that intellectually significant, I could just add an extra 1500 words and have a full length article. Because you write about 2000 words and they want more, so you get it longer and it’s getting to a normal article, you think well, the whole point – I thought the whole point is to have something succinct and sharp …. (#24)

## Failing to master the format

Our argument, so far, has emphasized how reliance on a relatively typified article format on the individual level facilitates reconciling various organizational contexts of research, including funding, collaboration with peers, communication with editors and reviewers as well as the writing process itself. Yet, this does not mean that producing such articles is trivial. Particularly because many STS journal submissions appear to follow a typical formula, unconvincing attempts tend to attract a particular form of critique by reviewers and editors, who themselves constantly alternate between reviewing and publishing journal articles. Besides documenting that even a typified format requires mastery and a degree of reflexivity, our informants’ accounts of experiencing such failure are analytically interesting also because they constitute a critical discourse about the perceived collective risks of STS journal publishing becoming overly formulaic.

A first common observation by scholars speaking from their perspective as editors and reviewers is a tendency for submissions to include literature sections that appear primarily designed to demonstrate familiarity with particular theoretical STS traditions and debates (#12, #50, and #62). The result is the impression of the author(s) ‘nodding’ at previous theoretical work to prove that they ‘speak STS’ (#50), but without developing or even using that work in any substantive way (#62). One editor speculated that such a practice goes along with a distinct use of digital search engines in the writing process:

[Through search engines like Google scholar] it becomes easier to search for papers on a topic of which you use keywords. And, thereby, easier to cite them. Just to show that you’re citing them regardless of whether you have any idea what they say. And then, … in many cases, the paper has what might be called ‘gestural theory’ or ‘elusive theory’, simply citing cryptically previous papers that use a particular theoretical concept, yet doing little with the concept. Now, of course, people might have done that decades ago, but it’s just easier now to give numerous citations that are thematically relevant, but almost as a substitute for doing something with the concept. … Perhaps to satisfy reviewers? … Or perhaps the author’s anticipation of the reviewers wanting to see a lot of citations. (#62)

Several informants in editorial positions (#12, #47, #62, and #69) observed that many submissions are built around an empirical case study – perhaps generated as part of a larger project – that is theorized by either superficial or overly derivative references to existing concepts that are mobilized as an analytical framework (#8 and #47). This creates the impression of manuscripts based on a specific argumentative formula of theory-applied-to-empirics, yet in ways that primarily lay bare the intent to facilitate the writing process by using said formula. One editor referred to ‘parroted analytical frameworks’ that often appear to be recycled from previous writing (#47), while another questioned the substance of the case studies themselves:

[There] is somewhat of a cookie cutter aspect to some of this work …, a certain kind of formula that gets deployed relatively [readily] and so allows people to kind of produce a lot. … Yeah, I mean, the case study stuff allows you to kind of pour content into a kind of existing mould, to a degree. It’s very much within the kind of the [scientization], I think, of STS. In terms of kind of more what I would consider to be kind of wide ranging, more, kind of, speculative essays, I don’t see that as much. (#8)

Another form of critique voiced by editors and referees focuses on ‘gaming’ strategies, such as attempting to facilitate the citeability of manuscripts through a practice reminiscent of search engine optimization (#62, #30, and #9). One editor observed a tendency for authors to coin a concept featured in the title or prominently presented in the opening sections to encourage uptake and reuse by readers, while the actual substance or novelty of the concept is unclear. Again, the effect is to show that authors have relied on, but failed, to master an unspoken formula that is widely deployed, raising questions about the originality and practical sustainability of STS journal publishing at large.

[You] read the manuscript and it is crafted in such a way that it attaches itself to literature and it does an analysis of something and it comes up with a new concept or it does something to innovate the field. But it’s, uhm, a little bit, almost gamey or almost like it feels like ‘we’re doing it because we can’, as opposed to because this is really something important that needs to be said or contributed …, making a big fanfare about launching a new concept. Then, the idea is that in the title you can have that new concept, then people can cite you and quote you for that concept. But then, when you look at what it actually is and how it purports to advance the literature, then it’s like there is not a whole lot there. … When the emphasis becomes on just getting through and just kind of tick all the boxes of what an article is supposed to look like, I get a bit frustrated. … I’m not kind of blaming people for that, it’s totally understandable. … People have no choice but to put this thing out and like well, ‘let’s just try it, let’s just send it, let’s just hand it over to these editors and to the reviewers. And then yeah, let’s hope that we get it in’. But I just feel that in a different system, such work might never see the light of day, or might just not even be sent in. (#9)

## Discussion

We have argued that the emergence of a typified journal article in STS is at least in part the result of increasingly quantified (self-)assessment practices in a highly competitive job market as well as a collective adaptation to grant-dependent, project-based work. Our analysis can be read as a contribution to recent writing on the governance of academic work through uncertainty, which has recurrently analyzed various tactics and workarounds academics use to deal with demands regarding the fundability, publishability, and originality of their work (Fochler & Sigl, 2018; Müller, 2014; Rushforth et al., 2019; Sigl, 2016). However, this literature has mostly focused on human actors and individual adaptation strategies. Our analytical focus has instead been on how a significant part of the scholarly communication system at large is restructured as researchers collectively react to novel expectations.

We wish to emphasize that we are not interested in romanticizing the publication culture of the past. STS in the 1980s and 1990s was, in some ways, comparable to an ‘old boys club’, with scholarly communication depending considerably on gate-keeping by a handful of influential journals. Neither do we mean to encourage a cliché version of experimental writing as an alternative. No one publication format is inherently more original than another, and imitating emphatically impressionistic or otherwise idiosyncratic writing in an attempt to revive the experiments of the 1980s could hardly be considered original. It also bears repeating that the template of the typical STS research article itself – on which we have ourselves relied – is not a trivial format to master and has many practical advantages. The convention of building an article around an empirical case study analyzed through the lens of established theory can, for example, be seen to have facilitated the broader influence of the field and possibly the empirical diversity of research topics. A significant amount of STS scholarship today is funded as part of a larger project connected to policy priorities, which in turn ensure an STS sensibility being brought into these settings. Yet, when systematically used on a large scale, the same typified article format will work to preclude forms of uncertainty from which STS as a field could otherwise benefit.

Let us elaborate on the implications in relation to the role of theory in STS journal articles. Theory is apparently often treated as a site of risk management by authors: You need to provide enough of it to ground your empirical work and legitimize it, but do so in a way that facilitates reception by peers and citations and yet without spending too much time on it. A simple problem besides efficiency concerns (as discussed above) may also be word limit, since tracing the genealogy of particular theoretical ideas and developing those ideas further often takes many pages, and theory sections in conventional articles always compete with the empirical analysis for space. We have already suggested that the resulting tendency toward incremental contributions (Ziman, 2000) and black-boxing of theoretical work is, to some extent, an inherent part of the development of research fields (Latour, 1987; Rheinberger, 1997; Whitley, 2000). But if a specific way of framing arguments achieves exclusive dominance for reasons of doability, there is a particular risk of formal or stylistic conservatism turning into intellectual conservatism. We believe that an approach of theory-as-compromise is actually particularly counterproductive in an interpretive field such as STS. In the natural sciences, research frontiers are both literary and empirical (Bazerman, 1988). While driven by questions arising from previous contributions, they ultimately break down with experimental results or novel empirical findings, if not always in strict synchronicity. In a field like STS, it is not primarily about proving theoretical framings right or wrong, but about their generative potential in enabling contributions that readers will find original, critical or, otherwise, inspiring. Exactly this potential to generate surprise on a conceptual level, however, is systematically diminished when a typical convention of how to frame arguments becomes too strong.

STS has, moreover, insisted that the epistemic aspects of knowledge are always intertwined with the specific historical subjectivities of its producers (Daston & Galison, 2010; Haraway, 1988). Academic labor markets have traditionally posed high demands on researchers’ personal discipline, incentivizing demonstrable productivity and intellectual specialization (Ben-David, 1977; Weber, 1992[1919]; Ziman, 2001). At the same time, it stands to reason that originality in a field like STS arises partly from an openness to subjectivities that are not from an early stage geared toward a specific idea of efficiency within sharply delineated research areas. Many of STS’s most influential thinkers had what would now be considered irregular trajectories – for example, switches from backgrounds in the natural sciences to interpretive scholarship or from activism to academia. This historical permeability has doubtlessly benefitted the field. The gradual emergence of a single typified format of output, however, could threaten the possibility of cultivating such hybridity and intellectual openness. Aside from the acceleration of article production it promotes and the concurrent career specialization required to compete with others, the typified article facilitates a quasi-industrial, labor-divided organization of the research process, where ‘grant acquisition’, ‘data collection’, and ‘writing up’ are conceived as separate tasks. This maps onto an implicit career structure that is based on a more differentiated and, simultaneously, more restrictive understanding of career progress – from a postdoctoral position that requires churning out publications to a professorship in which the actual working with material and development of arguments takes a back seat to more managerial tasks. It is unclear whether STS would have its current rich intellectual traditions, had it developed in a historical context in which such a career structure had already been firmly established.

## Supplemental Material

sj-docx-1-sss-10.1177_03063127221110623 – Supplemental material for Changing publication practices and the typification of the journal article in science and technology studies Supplemental material, sj-docx-1-sss-10.1177_03063127221110623 for Changing publication practices and the typification of the journal article in science and technology studies by Wolfgang Kaltenbrunner, Kean Birch, Thed van Leeuwen and Maria Amuchastegui in Social Studies of Science

sj-docx-2-sss-10.1177_03063127221110623 – Supplemental material for Changing publication practices and the typification of the journal article in science and technology studies Supplemental material, sj-docx-2-sss-10.1177_03063127221110623 for Changing publication practices and the typification of the journal article in science and technology studies by Wolfgang Kaltenbrunner, Kean Birch, Thed van Leeuwen and Maria Amuchastegui in Social Studies of Science
