# Supplementary material for: Changing publication practices and the typification of the journal article in science and technology studies
Source: Soc Stud Sci. 2022 Jul 28;52(5):758–82. doi: 10.1177/03063127221110623 (PMC9483190; doi:10.1177/03063127221110623)
Supplement: sj-docx-1-sss-10.1177_03063127221110623 – Supplemental material for Changing publication practices and the typification of the journal article in science and technology studies [file sj-docx-1-sss-10.1177_03063127221110623.docx]

**Appendix 1: Diversity of publication formats in STS journals**

**Table 1**

|  | **articles** | **comments** | **discussion pieces** | **notes & letters** | **reports & documents** | **reviews (books, media, exhibitions)** |
| --- | --- | --- | --- | --- | --- | --- |
| ***SSS*** |  |  |  |  |  |  |
| 1990 | 13 | 1 | 14 | 2 | 0 | 11 |
| 1991 | 18 | 0 | 11 | 5 | 0 | 8 |
| 1992 | 19 | 0 | 14 | 2 | 0 | 8 |
| 1993 | 15 | 0 | 4 | 4 | 0 | 7 |
| 1994 | 13 | 2 | 6 | 7 | 0 | 12 |
| 2015 | 33 | 6 | 2 | 0 | 0 | 3 |
| 2016 | 37 | 0 | 1 | 0 | 0 | 1 |
| 2017 | 37 | 0 | 3 | 0 | 0 | 2 |
| 2018 | 36 | 0 | 0 | 0 | 0 | 0 |
| 2019 | 38 | 0 | 0 | 0 | 0 | 0 |
| ***ST&HV*** |  |  |  |  |  |  |
| 1990 | 18 | 1 | 6 | 1 | 0 | 3 |
| 1991 | 19 | 3 | 4 | 0 | 0 | 5 |
| 1992 | 22 | 1 | 7 | 0 | 0 | 9 |
| 1993 | 23 | 3 | 3 | 3 | 0 | 12 |
| 1994 | 20 | 2 | 2 | 0 | 0 | 11 |
| 2015 | 36 | 1 | 0 | 0 | 0 | 5 |
| 2016 | 39 | 0 | 0 | 0 | 0 | 1 |
| 2017 | 40 | 4 | 0 | 0 | 0 | 1 |
| 2018 | 39 | 2 | 1 | 0 | 0 | 0 |
| 2019 | 40 | 2 | 1 | 0 | 0 | 2 |
| ***Minerva*** | |  |  |  |  |  |
| 1990 | 12 | 0 | 0 | 0 | 7 | 4 |
| 1991 | 12 | 0 | 0 | 0 | 4 | 4 |
| 1992 | 16 | 0 | 0 | 0 | 11 | 3 |
| 1993 | 13 | 0 | 0 | 1 | 6 | 4 |
| 1994 | 12 | 0 | 0 | 0 | 1 | 4 |
| 2015 | 17 | 0 | 0 | 1 | 0 | 3 |
| 2016 | 18 | 0 | 0 | 0 | 0 | 2 |
| 2017 | 21 | 0 | 0 | 0 | 0 | 1 |
| 2018 | 21 | 0 | 0 | 0 | 1 | 0 |
| 2019 | 24 | 0 | 0 | 0 | 0 | 2 |
| ***SaC*** |  |  |  |  |  |  |
| 1990 | 9 | 1 | 0 | 0 | 0 | 5 |
| 1991 | 17 | 0 | 0 | 0 | 0 | 9 |
| 1992 | 8 | 0 | 0 | 0 | 0 | 7 |
| 1993 | 16 | 0 | 1 | 0 | 0 | 8 |
| 1994 | 3 | 1 | 0 | 0 | 0 | 5 |
| 2015 | 22 | 0 | 0 | 0 | 0 | 7 |
| 2016 | 20 | 4 | 0 | 0 | 0 | 11 |
| 2017 | 19 | 3 | 0 | 0 | 0 | 13 |
| 2018 | 19 | 0 | 0 | 0 | 0 | 15 |
| 2019 | 21 | 0 | 3 | 0 | 0 | 4 |

Table 2

|  | **articles** | **introduced by reference to literature** | **based on empirical work** | **contains explicit methods section** | **historical data** | **document analysis** | **interviews** | **ethnographic work** | **quantitative analysis** |
| --- | --- | --- | --- | --- | --- | --- | --- | --- | --- |
| ***SSS*** |  |  |  |  |  |  |  |  |  |
| 1990 | 13 | 11 | 12 | 1 | 6 | 2 | 1 | 2 | 0 |
| 1991 | 18 | 15 | 18 | 8 | 4 | 8 | 2 | 1 | 4 |
| 1992 | 19 | 12 | 19 | 4 | 13 | 2 | 0 | 2 | 3 |
| 1993 | 15 | 15 | 15 | 1 | 8 | 3 | 3 | 1 | 0 |
| 1994 | 13 | 12 | 11 | 3 | 4 | 4 | 2 | 0 | 1 |
| 2015 | 33 | 26 | 27 | 10 | 6 | 2 | 7 | 9 | 2 |
| 2016 | 37 | 37 | 36 | 14 | 6 | 7 | 9 | 15 | 2 |
| 2017 | 37 | 37 | 34 | 13 | 6 | 9 | 9 | 9 | 1 |
| 2018 | 36 | 36 | 32 | 10 | 9 | 11 | 7 | 8 | 0 |
| 2019 | 38 | 38 | 38 | 18 | 3 | 10 | 8 | 16 | 1 |
| ***ST&HV*** |  |  |  |  |  |  |  |  |  |
| 1990 | 18 | 16 | 12 | 4 | 3 | 4 | 0 | 1 | 4 |
| 1991 | 19 | 12 | 15 | 9 | 1 | 3 | 4 | 3 | 2 |
| 1992 | 22 | 12 | 9 | 5 | 1 | 3 | 3 | 0 | 4 |
| 1993 | 23 | 17 | 9 | 3 | 4 | 3 | 3 | 1 | 1 |
| 1994 | 20 | 15 | 16 | 6 | 1 | 10 | 1 | 4 | 3 |
| 2015 | 36 | 36 | 34 | 22 | 1 | 10 | 10 | 5 | 4 |
| 2016 | 39 | 38 | 33 | 21 | 0 | 9 | 11 | 10 | 2 |
| 2017 | 40 | 38 | 35 | 15 | 5 | 11 | 10 | 6 | 4 |
| 2018 | 39 | 39 | 40 | 20 | 4 | 12 | 9 | 9 | 3 |
| 2019 | 40 | 40 | 38 | 17 | 2 | 0 | 12 | 12 | 1 |
| ***Minerva*** | |  |  |  |  |  |  |  |  |
| 1990 | 12 | 2 | 5 | 0 | 4 | 1 | 0 | 0 | 0 |
| 1991 | 12 | 2 | 8 | 1 | 5 | 2 | 0 | 0 | 1 |
| 1992 | 16 | 3 | 13 | 0 | 7 | 2 | 1 | 0 | 0 |
| 1993 | 13 | 3 | 11 | 0 | 10 | 1 | 0 | 0 | 0 |
| 1994 | 12 | 3 | 13 | 0 | 11 | 6 | 1 | 0 | 0 |
| 2015 | 17 | 17 | 19 | 7 | 4 | 4 | 4 | 2 | 4 |
| 2016 | 18 | 17 | 16 | 12 | 2 | 4 | 4 | 4 | 1 |
| 2017 | 21 | 20 | 20 | 12 | 4 | 5 | 5 | 2 | 4 |
| 2018 | 21 | 20 | 21 | 14 | 6 | 4 | 4 | 1 | 0 |
| 2019 | 24 | 24 | 21 | 16 | 2 | 5 | 5 | 2 | 5 |
| ***SaC*** |  |  |  |  |  |  |  |  |  |
| 1990 | 9 | 1 | 4 | 0 | 0 | 2 | 1 | 0 | 0 |
| 1991 | 17 | 5 | 11 | 1 | 2 | 8 | 1 | 0 | 0 |
| 1992 | 8 | 3 | 3 | 0 | 1 | 3 | 0 | 1 | 0 |
| 1993 | 16 | 6 | 15 | 0 | 6 | 6 | 1 | 1 | 0 |
| 1994 | 3 | 3 | 1 | 0 | 1 | 0 | 0 | 0 | 0 |
| 2015 | 22 | 21 | 20 | 11 | 2 | 4 | 9 | 5 | 0 |
| 2016 | 20 | 23 | 23 | 9 | 3 | 3 | 8 | 10 | 0 |
| 2017 | 19 | 22 | 20 | 13 | 1 | 11 | 7 | 4 | 1 |
| 2018 | 19 | 20 | 20 | 11 | 1 | 7 | 8 | 4 | 0 |
| 2019 | 21 | 21 | 24 | 15 | 1 | 6 | 9 | 7 | 0 |
