# Supplementary material for: Changing publication practices and the typification of the journal article in science and technology studies
Source: Soc Stud Sci. 2022 Jul 28;52(5):758–82. doi: 10.1177/03063127221110623 (PMC9483190; doi:10.1177/03063127221110623)
Supplement: sj-docx-2-sss-10.1177_03063127221110623 – Supplemental material for Changing publication practices and the typification of the journal article in science and technology studies [file sj-docx-2-sss-10.1177_03063127221110623.docx]

**Appendix 2: Scientometric methods**

The scientometric elements of this study draw on the in-house version of the WoS database at the Centre for Science & Technology Studies (CWTS) at Leiden University. This database has significantly more capabilities and content when compared to the online, desktop version. Aside from containing comprehensive bibliographic information, such as formal article characteristics, author affiliation, and references, the database is connected to additional datasets that allow us to conduct meta level research, for example on country and field data over a longer period of time, or on networks of research performing organizations.

The analysis presented in this paper focuses on the publication activity of scholars in STS journals for the period 1981-2018. The starting year was chosen for the practical reason that our database provides largely consistent coverage from 1981 onwards. Naturally, the attempt to delineate STS as a field is inherently problematic, since by defining the ‘in’, one immediately defines an ‘out’. Our approach in selecting relevant journals in WoS has been to rely on an inventory of STS periodicals that was until recently provided on the website of the Society of Social Studies of Science. Drawing on this resource, we initially identified 25 journals as relevant to our study. Of these, however, 7 are not covered in WoS, and thus not amenable to scientometric analysis. We consider the remaining 18 journals as representative of STS journal publishing, but we also recognize that STS articles are published in other journals not covered here.

Table 3

|  | Journal | WoS data available as of | Included in our analysis (Y/N) |
| --- | --- | --- | --- |
| 1 | *Catalyst: Feminism, Theory, Technoscience* | - | N |
| **2** | ***Computer Supported Cooperative Work (CSCW): The Journal of Collaborative Computing and Work Practices*** | **2008** | **Y** |
| **3** | ***Configurations: A Journal of Literature, Science, and Technology*** | **1995** | **Y** |
| **4** | ***East Asian Science, Technology and Society: An International Journal*** | **2018** | **Y** |
| 5 | *Engaging Science, Technology, and Society* | - | N |
| **6** | ***Journal of Responsible Innovation*** | **2019** | **Y** |
| **7** | ***Minerva: A Review of Science, Learning and Policy*** | **1979** | **Y** |
| **8** | ***New Genetics and Society: Critical Studies of Contemporary Biosciences*** | **1999** | **Y** |
| 9 | *Prometheus: Critical Studies In Innovation* | - | N |
| **10** | ***Public Understanding of Science*** | **1997** | **Y** |
| **11** | ***Research Policy: Policy, management and economic studies of science, technology and innovation*** | **1980** | **Y** |
| 12 | *Science & Technology Studies* | - | N |
| **13** | ***Science and Engineering Ethics*** | **1998** | **Y** |
| **14** | ***Science and Public Policy*** | **2009** | **Y** |
| **15** | ***Science as Culture*** | **1987** | **Y** |
| **16** | ***Science Communication*** | **1994** | **Y** |
| **17** | ***Science in Context*** | **1994** | **Y** |
| **18** | ***Science, Technology, & Human Values*** | **1980** | **Y** |
| **19** | ***Social Epistemology: A Journal of Knowledge, Culture and Policy*** | **2011** | **Y** |
| **20** | ***Social Studies of Science*** | **1980** | **Y** |
| 21 | *Tapuya: Latin American Science, Technology and Society* | - | N |
| **22** | ***Technology Analysis and Strategic Management*** | **1994** | **Y** |
| 23 | *TECNOSCIENZA: Italian Journal of Science & Technology Studies* | - | N |
| **24** | ***The Journal of Cultural Economy*** | **2011** | **Y** |
| 25 | *Valuation Studies* | - | N |

For the 18 selected journals, we have collected publication data from the CWTS in-house version of WoS. The analysis focuses on a number of aspects. Firstly, bibliographic data can be analyzed to study longitudinal changes in formal features of publications, such as average page length and number of references. Secondly, access to author information allows us to monitor whether the number of collaborating authors increases or remains stable over time. Scholarly collaboration can be further studied by focusing on changes in the number of distinct institutional affiliations provided in the contact details of publications. We also provide an analysis of the outgoing references of the articles in our dataset, focusing on how many of these references relate back to other publications in WoS. This allows for inferences about what types of materials are cited by authors publishing in STS journals, and how important WoS is to the field and over time. A caveat is that reference data in earlier years is in several journals less consistently formatted, making our dataset somewhat less reliable before ca. 1990.
